# Understanding the impact of an AI-enabled conversational agent mobile app on users’ mental health and wellbeing with a self-reported maternal event: a mixed method real-world data mHealth study

**DOI:** 10.3389/fgwh.2023.1084302

**Published:** 2023-06-02

**Authors:** Becky Inkster, Madhura Kadaba, Vinod Subramanian

**Affiliations:** ^1^ Department of Psychiatry, University of Cambridge, Cambridge, United Kingdom; ^2^ Wysa Inc., Boston, MA, United States

**Keywords:** maternal mental health and wellbeing, artificial intelligence, psychotherapy, depression, conversational agent (CA), chatbot

## Abstract

**Background:**

Maternal mental health care is variable and with limited accessibility. Artificial intelligence (AI) conversational agents (CAs) could potentially play an important role in supporting maternal mental health and wellbeing. Our study examined data from real-world users who self-reported a maternal event while engaging with a digital mental health and wellbeing AI-enabled CA app (Wysa) for emotional support. The study evaluated app effectiveness by comparing changes in self-reported depressive symptoms between a higher engaged group of users and a lower engaged group of users and derived qualitative insights into the behaviors exhibited among higher engaged maternal event users based on their conversations with the AI CA.

**Methods:**

Real-world anonymised data from users who reported going through a maternal event during their conversation with the app was analyzed. For the first objective, users who completed two PHQ-9 self-reported assessments (*n* = 51) were grouped as either higher engaged users (*n* = 28) or lower engaged users (*n* = 23) based on their number of active session-days with the CA between two screenings. A non-parametric Mann–Whitney test (M–W) and non-parametric Common Language effect size was used to evaluate group differences in self-reported depressive symptoms. For the second objective, a Braun and Clarke thematic analysis was used to identify engagement behavior with the CA for the top quartile of higher engaged users (*n* = 10 of 51). Feedback on the app and demographic information was also explored.

**Results:**

Results revealed a significant reduction in self-reported depressive symptoms among the higher engaged user group compared to lower engaged user group (M–W *p *= .004) with a high effect size (CL = 0.736). Furthermore, the top themes that emerged from the qualitative analysis revealed users expressed concerns, hopes, need for support, reframing their thoughts and expressing their victories and gratitude.

**Conclusion:**

These findings provide preliminary evidence of the effectiveness and engagement and comfort of using this AI-based emotionally intelligent mobile app to support mental health and wellbeing across a range of maternal events and experiences.

## Introduction

Parenthood is a transition that can pose significant challenges to mental and physical health across the maternal spectrum, including pre-conception, antenatal period, and during or after giving birth (1–10).

Perinatal mental health disorders are common (11). In the United Kingdom (UK), perinatal mental health problems can affect 10%–20% of women either during pregnancy or within one year of giving birth (12). According to the American Pregnancy Association and Postpartum Support International, approximately 70%–80% of new mothers experience negative feelings after giving birth (13). For certain demographic groups this is higher, for example, up to 60% for adolescent mothers with a low income (14, 15). Depression during pregnancy and the postpartum period is associated with multiple poor outcomes for parental well-being and childhood development (7, 16–18).

Maternal mental health is a global public health and economic challenge (19–22). The estimated accumulated national economic cost of perinatal depression and anxiety in the UK is £6.6 billion (11). While treatments for maternal mental health care exist, research suggests that implementation can be challenging and variable (23).

Technology could play a significant role in addressing barriers to care for maternal mental health (24, 25). The acceptability, feasibility and effectiveness of perinatal depression digital interventions have been evaluated in various pilot studies, randomized controlled trials (RCTs) and systematic reviews (26–36). While maternal mental health digital technology could help improve accessibility, offer timely psychosocial support, as well as potentially improve the quality of information being collected, much more research is needed in this area to carefully examine its potential benefits vs. its limitations (37). A study that published recommendations based on user feedback to inform future development of digital maternal mental health support included the proposal of adding an Artificial Intelligence (AI) chatbot (38). Using dialogue-led personalized tools, AI-based Conversational agents (CAs; chatbots) could potentially facilitate effective and safe guided conversations and collect information beyond limited survey questions and self-guided sessions.

AI systems using CA have already been developed to provide medical information to support child physical health for new mothers (39–41). In more recent years, proposals have emerged on how AI could play a distinctive digital role in supporting maternal mental health and wellbeing (42). A clinical trial that randomized women during their birth hospitalization to either “chatbot plus treatment as usual” or “treatment as usual” (TAU) reported that many participants used the chatbot at least once every 2 weeks and that most users reported medium or high satisfaction with the CA AI system (43). The authors also reported that most participants reported medium or high degrees of therapeutic alliance and acceptability (43). Related to these findings, an additional RCT publication evaluated the effectiveness of the automated CA on changes in symptoms of anxiety and depression. The authors reported that at the 6-week postpartum follow-up there were no statistically significant group differences between the “chatbot use” or “TAU” (44). A later related publication reported that the CA intervention group (“WB001+ TAU”) had a significant reduction in depression scores as compared to a TAU-only control group 6-week postpartum after birth hospitalization (45).

A pre-pilot development and usability study examining an AI system involving a cohort of enrolled Kenyan pregnant women and new mothers reported that most women submitted at least three mood ratings, sent at least one message to the AI system and that approximately a third of women engaged beyond registration (46). Most AI users reported a positive attitude and having trust in using the AI system and that life changes were attributed to using it and reported an estimate that using the alpha version of the AI system may have improved mood (46).

A different approach using a supervised machine learning CA for perinatal mental healthcare was proposed by authors that could be an effective approach for monitoring the mental health status of perinatal women in real time while collecting user health data. The authors analyzed the 31 characteristics of 223 samples and trained a supervised machine learning model to determine the anxiety, depression, and hypomania index of perinatal women (47).

While this literature shows some degree of initial promise, much more research is required to determine how CA AI systems can safely support maternal mental health and hence the motivation to perform our study as a contribution toward furthering the understanding AI's potential role in maternal mental health.

In our study, we examine the use of an AI-based emotionally intelligent mobile app (“Wysa”) aimed at building mental resilience and promoting mental well-being using a text-based CA. Previous studies evaluating Wysa have shown significant reductions in depressive symptoms (48–50). The conversation-based tools and techniques encourage users to manage their anxiety, energy, focus, sleep, relaxation, loss, worries, conflicts, and other concerns. Wysa responds to emotions that a user expresses and recommends evidence-based self-help tools and techniques such as Cognitive Behavioural Therapy (CBT), Acceptance and Commitment Therapy (ACT), Dialectical Behaviour Therapy (DBT), motivational interviewing, positive behavior support, behavioral reinforcement, mindfulness, and guided micro actions that encourage users to build emotional resilience skills.

Our study has two objectives: (1) To examine the effectiveness of Wysa by comparing changes in self-reported depressive symptoms between higher vs. lower engaged groups (*n* = 51) involving users that self-reported a maternal event, and (2) To perform a qualitative analysis to understand the themes being raised for a subset of user messages provided by higher engaged users (*n* = 10). This study also discusses *post hoc* observations, clinical implications, and other research implications derived from our study.

## Methods

### Study design and participants

The study duration occurred between February and September, 2019 (pre-pandemic). The participants were initially selected from a pool of real-world users (*N* = 380,500 users) who used the Wysa app during the study period and who submitted any of the maternal event keywords during their conversation with the CA (*n* = 5,373). Individuals reported at least once about an ongoing maternal event in response to one of these messages: (1) “Tell me about any recent major changes or events in your life. It could be something stressful or even a good change like moving home or getting a new job.”, (2) “Take a few deep breaths. Tell me the first thought on your mind?”, (3) “I’m here for you. What exactly happened?” (4) “Okay let's talk about that. Go on”, (5) “I understand. Is there more?”. Multiple example screenshots of the Wysa app that match the time period of this study can be found in Supplementary Material S1.

A subgroup of *N* = 2,037 users (2,037 out of 5,373 active users mentioned above) were then grouped into four maternal event categories: Pre-pregnancy, Pregnancy, Perinatal, and Postpartum, which are defined in Table 1. This categorization was used by the researcher for the thematic qualitative analysis (testing Objective 2) to assist in making observations that were categorised into the different maternal event stages (pre-pregnancy and pre-conception, pregnancy, perinatal, postpartum). Furthermore, eligibility (inclusion and exclusion) criteria was applied to determine user enrolment into the study, which is listed in Table 2 and Supplementary Multimedia Appendix A, the latter of which shows the sampling method used for this study.

**Table 1:** Users were grouped into the following categories based on their self-reported maternal events.

Maternal event categories	Description
Pre-Pregnancy or Pre-Conception	Period prior to conceiving or pregnancy
Pregnancy	Period up-to 22 weeks of pregnancy
Perinatal	Period from 22 weeks of pregnancy until first month of childbirth
Postpartum	Period >1 month to ≤1 year post childbirth

**Table 2:** The following inclusion and exclusion criteria were defined to determine participant eligibility.

Inclusion criteria	Exclusion criteria
• Self-reported maternal event • Female users • Android and iOS smartphone users • Wysa app usage during study period (start-and-end dates inclusive)	• Users who were tagged as male • Users who were tagged as gender ambiguous • Users who talked about maternal-event of a third party • Users whose records indicated more than one year after child birth

A final sample of *n* = 10 users were included in the qualitative analysis. The selection method used a non-probability-based sampling to select 10 users who had an engagement density of greater than 75%. Furthermore, after additional eligibility screening was applied based on the criteria of completing the mental health self-reported depressive symptoms assessment questionnaire at two time points (defined below in the Data Analysis Section “Effectiveness Analysis” section) a final sample size of *n* = 51 users were included in the Effectiveness Analysis (testing Objective 1 using a statistical analysis approach).

### Instruments (measures)

The Patient Health Questionnaire 9 (PHQ-9) was used to measure self-reported depressive symptoms at baseline and follow-up. This validated self-report questionnaire (51) consists of nine DSM-IV criteria (i.e., questions) and the participant answers (i.e., scores) each of those on a scale from 0 to 3 (“0” = not at all, “1” = several days, “2” = more than half the days, “3” = nearly every day), whereby the severity of depression is measured on the final score aggregated across the questions and range from 0 to 27 points. It is interpreted using these cut-off points: Scoring between 0 and 4 points indicates minimal depression, 5 and 9 points indicates mild depression, 10 and 14 points indicates moderate depression, 15 and 19 points indicates moderately severe depression, and 20 or more points indicates severe depression. The PHQ-9 assessments were voluntary, and users were notified once every two weeks.

### Data collection

The app repository was queried for a predefined set of maternal event keywords (Table 3).

**Table 3:** The following inclusion and exclusion criteria for maternal event keywords were used to query the app repository for relevant user records event keywords.

Keywords included	Keywords excluded
*partum* OR ppd OR preg* OR natal OR *birth OR miscar* OR delivery OR matern* OR patern* OR birthing OR parturition OR ivf OR *fertil* OR doula OR epidur* OR “water break” OR “water broke” OR abort* OR “mid wife” OR midwife	*baby* OR child* OR labor OR labor OR mum OR mom OR mother* OR *born

Certain keywords were excluded that were considered to induce many false positives within the data (e.g., “born” outside of a maternal-related context, such as “I wish I was never born”). Messages (“user records”) with included keywords were extracted along with the user's app engagement information. The user records were cleared for any inadvertently submitted Personal Identifiable Information (PII) using Wysa's proprietary PII detection and redaction algorithm. The de-identified user records were tagged manually for the maternal event categories. The user records were also tagged for gender: “Female”, or “Male” or “No Tag Available” when gender-related information was not available.

### Data analysis

A mixed-methods quantitative and qualitative approach was used to evaluate our two study objectives on efficacy and engagement, respectively.

### Effectiveness analysis (objective 1)

The Patient Health Questionnaire (PHQ-9) was used to measure self-reported depressive symptoms at two time points. The sample size was comprised of 51 users who completed the PHQ-9 at least two weeks apart but not more than 5 weeks apart, and scored more than 3 in the PHQ-2 (the first 2 questions of the PHQ-9) (51). The self-reported PHQ-2 cut-off was set at >3 (minimum of 4, maximum of 6). The Self-Reported PHQ-9 was set at greater or equal to 5 (minimum of 5, maximum of 27, overall PHQ-9 score of 27; see Instruments measures section for more context).

Two comparison groups were identified: (1) a higher engaged user group (*n_h_*_ _= 28) and (2) a lower engaged user group (*n_l_*_ _= 23), based on the number of “session-days” (the days the users engaged with the CA between the two PHQ-9 screening days).

“Engagement density” is a normalized measure calculated for each user defined as the number of active session-days with the CA in-between the two PHQ-9 assessments divided by the available days between the two screenings. Users whose engagement density was ≥0.4 were grouped as “higher engaged group” and those with engagement density <0.4 were grouped as “lower engaged group”. A 40% engagement density translated to 14 active days of AI-enabled CA sessions over a 35-day period.

The average change in depressive symptoms (first PHQ-9 assessment score minus the second PHQ-9 assessment score) was compared between the higher vs. lower engagement groups. A two-tailed, 5% significance Mann–Whitney test was used to test the statistical significance of the difference in average change of symptoms between the two groups. The effect size was measured using the nonparametric common language effect size (CL) (52, 53).

We further examined the data related to objective 1 to assess the clinically meaningful impact from the quantitative results using the categories defined by the PHQ-9 to discuss the clinical implications.

Furthermore, a *post hoc* analysis was performed to explore changes in the severity thresholds between the two comparison groups across increasing PHQ-9 severity (at ≥10, 15 and 20 intervals). Depressive symptom reductions were measured for statistical significance (M–W test) and effect size (CL) but not considered as actually significant due to the exploratory, *post hoc* nature and lack of correction for multiple testing.

### Engagement analysis (objective 2)

A qualitative analysis examined behaviors exhibited by a subset of higher engaged users with maternal events based on their conversations with Wysa. A Braun and Clarke thematic analysis (54, 55) was performed on free-text responses from users (*n* = 10) who had an engagement density of greater than 75% based on their conversations (i.e., an anonymized sample of 216 free-text conversational snippets) with the AI CA. The main themes and subthemes, derived from the coding and analysis, helped understand users' expectations, experience and engagement. Prevalence of a theme was measured based on number of instances and number of responding users.

### Ethics

Wysa is publicly available as a mobile application (android and iOS). The Conversational Agent (CA) is freely available and has been designed to prioritize safety, privacy and security-by-design. No user registration is required and no Personal Identifiable Information (PII) is asked for during app use. This provides users a private, anonymous space encouraging them to manage their mental well-being in a self-help context (56). All content and tools within the app are reviewed and validated by the Wysa clinical and safety teams. As the study involved analyzing real-world data from an anonymous nonclinical population, it was exempt from registration in a public trial registry (according to OHRP guidelines). Users voluntarily downloaded the app after having consented to the app Terms of Service and Privacy Policy (57, 58). For ethical and privacy reasons, the authors did not have access to all the user messages. Only minimal and limited conversation data at specific chat endpoints were used. The dataset was anonymised by redacting any inadvertent identifiers. User data was adequately secured according to the organization's privacy, security and safety policies. The organization's compliance and privacy officer is author VS who audited the study dataset for safety, privacy and security compliance prior to research use.

## Results

### Effectiveness analysis (objective 1)

**Figure 1:**
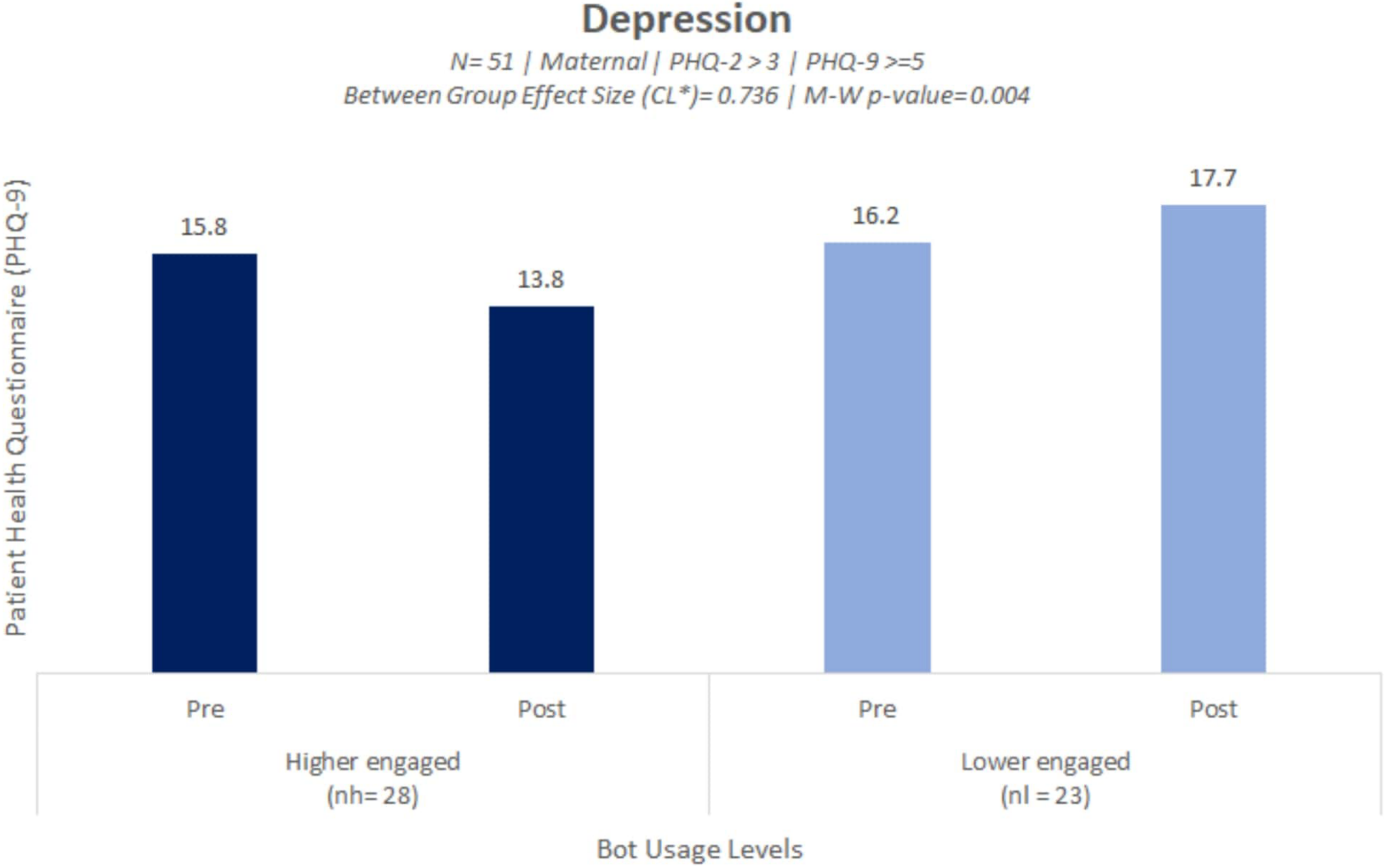
Bar plot illustrating a significant reduction in symptoms of self-reported depressive symptoms amongst the higher engaged user group compared to the lower engaged user group.

The effectiveness statistical analysis was performed to test for group level differences in depressive symptom scoring between high vs. low engagement group of users. The statistical analysis revealed that the high engaged user group showed a significant depressive symptom reduction compared with the lower engaged user group (*p* = .004) [Figure 1, also refer to Table 4 for more information including a breakdown of PHQ-9 score averages and standard deviations (SD) for each group pre and post timepoints]. The effect size was high (large) (0.736) (also see Table 4), which is roughly equivalent to a high Cohen d (0.89) (52, 53). There was no statistical difference in average PHQ-9 baseline scores between the higher and lower engaged groups. The high engagement group at baseline had a PHQ-9 minimum of 6 and a maximum of 24 and at follow-up had a PHQ-9 minimum of 6 and a maximum of 27. The low engagement group at baseline had a PHQ-9 minimum of 9 and a maximum of 24 and at follow-up had a PHQ-9 minimum of 9 and a maximum of 27.

**Table 4:** The high engaged user group showed a significant reduction in depressive symptoms compared with the lower engaged users group (*p* = 0.004).

Users with self-reported PHQ-2^a^ > 3 & PHQ-9 > 5	Number of users (*N*)	First PHQ 9 score (SD)	Second PHQ 9 score (SD)	Mean difference	Mann–Whitney *U* (*p* value^c^)	Effect size (CL)^b^
Higher engaged users (*n_h_*)	28	15.8 (6.0)	13.8 (5.4)	2.00	170 (0.004)	0.736
Lower engaged users (*n_l_*)	23	16.2 (4.2)	17.7 (4.6)	−1.50	–	–

^a^ PHQ-2, Patient Health Questionnaire-2.

^b^ CL, common language effect size.

^c^ 95% significance.

The decrease in PHQ-9 score for the higher engagement group was indicative of a shift in clinical improvement from “moderately severe” depression at baseline into the “moderate depression” category at follow-up (refer to Methods Section about PHQ-9 scoring assessment procedures). There was no clinical shift between scoring categories for the lower engagement group and at both time points the lower engagement group average PHQ-9 scores remained within the “moderately severe depression” range.

### *Post hoc* quantitative analysis

**Figure 2:**
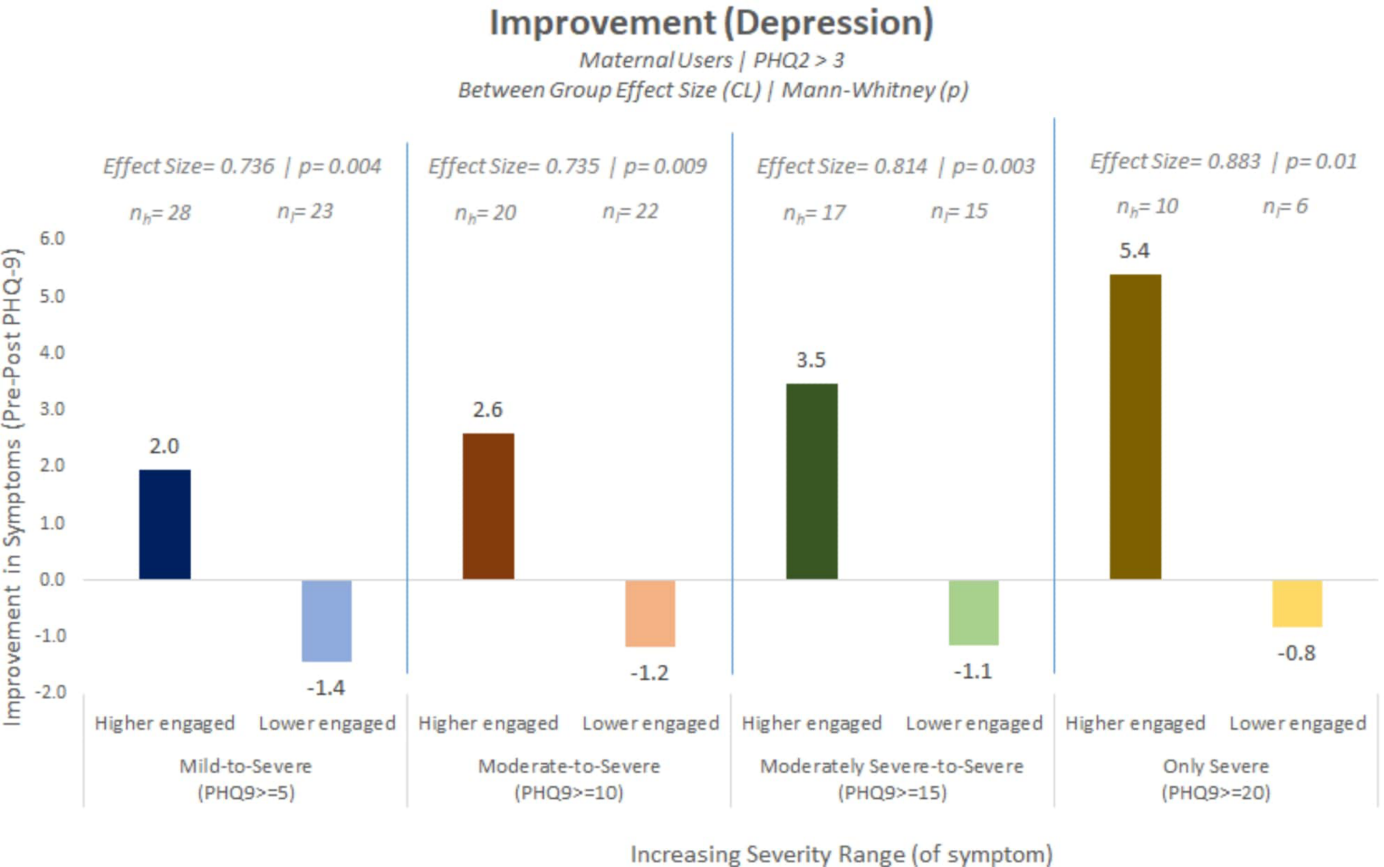
*Post hoc* analysis showing significant reductions in depressive symptoms among higher engaged users compared to lower engaged users across multiple PHQ-9 severity cut-off thresholds.

As a *post hoc* analysis, we explored depressive symptom reductions between the groups across increasing PHQ-9 severity. Significant reductions were found among higher engaged users compared to lower engaged users as the severity increased in that the effect was stronger for higher PHQ-9 scoring category thresholds indicating the effect was stronger for more severe depression. Depressive symptom reductions were seen with large effect sizes (0.735–0.883) at PHQ-9 ≥ 10, 15 and 20 intervals as can be seen in Figure 2.

### Engagement analysis (objective 2)

We report results derived from qualitative insights into the behaviors exhibited among higher engaged maternal event users (*n* = 10; engagement density <75%) based on their conversations (an anonymized sample of 216 free-text conversational snippets) with the AI CA. Five main behavioral themes emerged: (1) Concern, (2) Support, (3) Reframe, (4) Hope, and (5) Victory. More detailed information about these themes is reported in Table 5. For the thematic maps of themes and sub-themes, and user messages, please refer to Supplementary Multimedia Appendix B. In brief, some users used the app to explore and express their feelings or concerns, or were more critical of themselves or others, or actively and repeatedly used sleep, relaxation and anxiety related tools or techniques to manage their emotional states, or users completed CBT and reframed their negative thoughts as they shared their small victories and gratitudes during their self-created personalized self-help journey. Notably, none of the users sought help from the CA about their maternal health related matters. This was not an identified theme or sub-theme.

**Table 5:** Five main behavioral themes emerged from the qualitative analyses of free-text responses from higher engagement AI users.

Themes	Subthemes
“Concern”	This theme brings out the user’s emotional state as they share their issues, worries, negative thoughts and emotions. Users mentioned multiple life stressors, for example, expressing fear about an underlying health condition or fear of getting pregnant or generally fearful of things going around them. Users were often found to be self-critical and expressed an underlying anguish or frustration over their relationship, work-life challenges or their emotional situation.
“Support”	This theme captures instances where users came looking for well-being support. It also included those who expressed their frustration for the lack of support available outside and how they appreciated the CA for the support provided. User messages highlighted a continuous search to find support mechanisms, to seek love and understanding, to seek help for managing emotions and leading their day-to-day life.
“Reframe”	The most used CBT technique was reframing thoughts. Reframing helps the user to use CBT techniques to consider their thoughts from a different perspective and then to replay it to reinforce it. Users also identified and expressed possible solutions that they could work on to manage their issues. Some users used the CA to frame messages that they wanted to convey and to confront their relationship challenges.
“Hope”	Users found comfort in sharing their hopes and intentions about their well-being. They expressed hope to achieve some of their objectives. In the process they expressed goals for themselves and also that involved others.
“Victory”	Users motivated themselves to take control and action. They also shared their sense of achievement and positive activities. Users expressed their gratitude about key events, for tasks undertaken, about those close to them, about their well-being and including objects/tools.

### Additional qualitative observations

These users also provided feedback on sessions within the app with overall sessions seeing 97% of users with high or mid-satisfaction. The user's geographical zone location was derived from the zone related to their smartphone. Participants were mainly located in North America, with a small minority located in Europe. Relationship status was inferred from conversation snippets, which showed that most of the participants (50%) were single and only 10% were married.

Stressors experienced by the users were also identified with key concerns being about relationships (100% of users, included marriage, parents, trauma, break-ups), financial distress (60% of users, included worries about savings and money), work (20% of users, work-life balance, coworker discomfort), life (30% of users, including feelings of loneliness, worthlessness, disinterest), and physical health (10% of users, included chronic illness).

## Discussion

Technology's role in supporting mental health and wellbeing is increasingly evidenced. Our study adds to this literature showing how an AI-enabled CA can offer emotional support for maternal mental health and wellbeing. Given evidence that maternal depressive symptoms have increased during the COVID-19 pandemic (59, 60) our pre-covid study is important and requires follow-up to validate our preliminary findings.

### Principal findings

This study evaluated the effectiveness of an AI-enabled CA-based mental health and wellbeing app on reducing depressive symptoms for users who reported maternal events. We found a significant reduction in self-reported depressive symptoms among the higher engaged user group compared to the lower engaged user group with a high effect size. This reduction in PHQ-9 score for the higher engaged group was indicative of a shift in clinical improvement from “moderately severe depression” to “moderate depression” at follow-up. A *post hoc* analysis showed that reductions in depressive symptoms were observed across all PHQ-9 severity score thresholds for the higher engaged group, with an observed increasing effect size as severity of symptoms increased.

This study also performed a qualitative analysis to examine user engagement, which revealed five thematic behaviors: Concern, Support, Reframe, Hope and Victory. Some users used the app to explore and express their feelings or concerns. Some were more critical of themselves or others. Some users actively and repeatedly used sleep, relaxation and anxiety related tools or techniques to manage their emotional states. Some users completed CBT and reframed their negative thoughts as they shared their small victories and gratitudes during their self-created personalized self-help journey.

None of the users asked for support for their maternal event or maternal health matters. Instead, users messaged and engaged with the CA about their emotions and the stressors they were experiencing. This could suggest that users were aware of the intended purpose of the well-being app. The overall in-app feedback indicated comfort of using a digital mental health and wellbeing CA for support.

### Comparison with existing literature

We compared our findings with the existing literature focusing on publications that assessed the effectiveness of using CA AI systems for reducing maternal mental health depressive symptoms. Two publications of this type were identified (as this is a nascent research area).

An RCT study (44) was identified that evaluated the effect of an automated CA on postpartum mental health using three questionnaires, including the PHQ-9. The authors reported no significant difference between the “CA intervention group” and the “treatment as usual (TAU) control group” between baseline (after giving birth) and 6 weeks postpartum. That study differed from our study in multiple ways. Our study used a real-world anonymous remote setting approach to enrol users and we included a wide range of maternal events (pre-pregnancy and pre-conception, pregnancy, perinatal, postpartum) whereas the RCT study (44) took place in hospital settings with stringent recruitment eligibility criteria. The studies also differ by control group criteria, study durations, sample size, and differing PHQ-9 cut-off thresholds. The RCT reported low baseline PHQ-9 mean scores (intervention and control group average PHQ-9 scores of 4.6 and 3.3, respectively) and similarly low at 6-week follow-up (intervention and control groups, 3.1 and 3.1, respectively) whereas our study showed much higher group mean PHQ-9 scores (Table 4). It could be possible that differences in study designs, PHQ-9 score severity, and durations of questionnaire assessments are, in part, explaining differences in statistical findings.

Another RCT (45) [which might be closely related to the other RCT (44)] evaluated an automated CA on postpartum mental health using two mental health questionnaires, including the PHQ-9, and reported that the CA intervention group (“WB001+ TAU participants”) had a statistically significant reduction in depressive symptom scores compared to the TAU-only control group between baseline after giving birth and 6 weeks postpartum (45). The RCT reported similarly low PHQ-9 mean scores at baseline and 6-week follow-up (scores below 5). It is not possible to compare our study findings with this publication (45), however, as the methodology used is not described.

To our knowledge these two maternal mental health publications are the only literature currently available for direct comparison of statistical findings on CA AI system effectiveness using the PHQ-9 to assess changes in depressive symptoms. We were unable to compare efficacy with a third study (46) as that pre-pilot study did not include two timepoints for the PHQ-9 for various reasons. For example, the depression screening was too long to administer on a repeating basis, the researchers wanted to avoid frustrating users and distracting them from potential engagement with the intervention (46).

### Clinical implications

For the higher engagement group, the statistically significant decrease in PHQ-9 score was indicative of a clinical shift from “moderately severe depression” at baseline into the “moderate depression” category at follow-up. In contrast, for the lower engagement group there was no clinical shift between depressive symptom categories (it remained as “moderately severe depression”). If the higher engagement group used Wysa for a longer duration than two weeks it could be hypothesized that this would further reduce symptomology bringing users “below caseness”, which is defined by NHS IAPT services as being in either “minimal” (PHQ9 score of 0–4) or “mild” (PHQ9 score of 5–9) categories (61).

Our *post hoc* observations showing a reduction in depressive symptoms across all PHQ-9 severity score thresholds for the higher engaged group supports a prior meta-analysis, which showed that patients with more severe depression at baseline had at least as much clinical benefit from low intensity interventions as less severely depressed patients, inferring that low intensity interventions could be offered to more severe symptom groups (62).

In clinical settings, AI-enabled CA agents could potentially enhance health information systems to capture the right measurements at the right time and offer the right support in a timely manner, which could help to alleviate the burden of data collection by health-care workers in healthcare settings (63). This technology could also potentially support early detection given that many postnatal depression cases are undiagnosed (30). This is important in low-and middle-income countries (given the lack of trained professionals); however, even in countries where trained professionals are more readily available communication gaps in identifying concerns remain an issue (64), which is where an AI-enabled CA agent facilitate better communication.

There is much potential for using CA-based support for maternal mental health and wellbeing, but this will require more in-depth research and must ensure that safety and privacy protects users, especially given ongoing global events related to reproductive justice (65) and the role of technology companies (66). The mental health and wellbeing CA examined in our study prioritizes privacy and security by design and default. Two recent Mozilla Foundation reports found that reproductive health apps (67) and most digital mental health and wellbeing apps (68) investigated were problematic in terms of data protection and privacy with their report showing that Wysa was only 1 of 2 digital mental health and wellbeing apps to pass their privacy investigation (68).

In terms of generalizability, users came from 150 global time zones and both android and iOS versions of the app were used. Furthermore, this study included individuals who self-reported experiencing a wide range of maternal events that had an impact on their mental health and wellbeing. This helps to highlight the importance of offering AI-enabled CA support to wider demographics who need emotional support for different reasons with different needs and preferences (48–50).

This study has several limitations. As RCTs are typically regarded as the “gold standard” for evaluating the efficacy of interventions, a lack of controlled settings could lead to non-handling of biases. Our study has no way of knowing whether demographics were balanced across groups (e.g., diagnoses, other psychological support they might have sought, etc. could impact the analysis). While the PHQ-9 scores are indicative of depressive symptomatology, scores do not confirm or refute the presence of depression. Our study only collected PHQ-9 scores from two time points close together and was not able to follow-up on later outcomes. It did not use other assessments such as EPDS, which could be more appropriate to check concordance and discordance (69). This study used small, unbalanced comparison groups with no use of a treatment-as-usual control group. Users had voluntarily used the app and were likely to have high readiness to explore self-care tools. There may be human bias in labeling data for analysis, such as gender ambiguity, given the app doesn't capture gender for privacy reasons.

Overall, this study demonstrates that an AI-enabled CA-based support can play an important role in reducing depressive symptoms and offering support across diverse maternal events. It adds to a nascent yet growing literature demonstrating the acceptability and comfort of using AI-enabled CA-based digital mental health and wellbeing apps for emotional support.

## Data Availability

The raw data supporting the conclusions of this article will be made available by the authors, without undue reservation.
