# Supplementary material for: Understanding the impact of an AI-enabled conversational agent mobile app on users’ mental health and wellbeing with a self-reported maternal event: a mixed method real-world data mHealth study
Source: Front Glob Womens Health. 2023 Jun 2;4:1084302. doi: 10.3389/fgwh.2023.1084302 (PMC10272556; doi:10.3389/fgwh.2023.1084302)

**Supplementary Material 1**

Images are example screenshots from the AI-enabled Wysa app, which participants could access during the study period time in 2019.


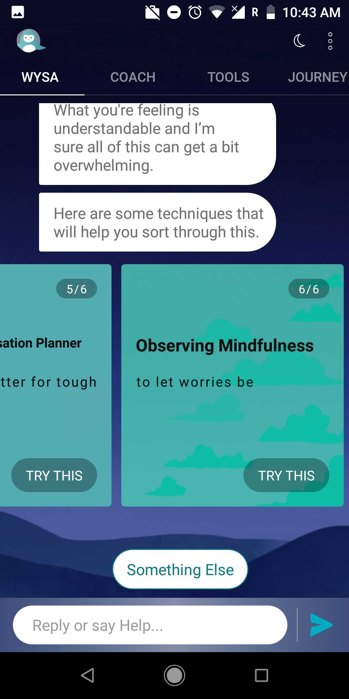


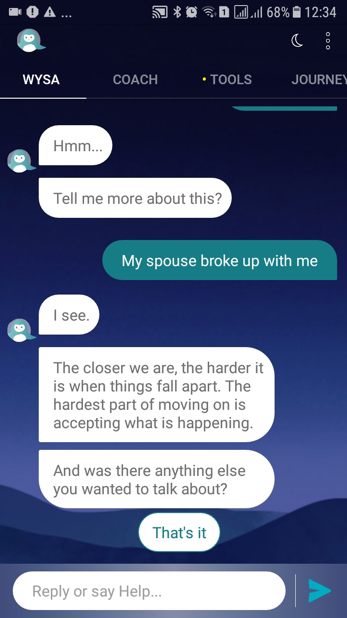


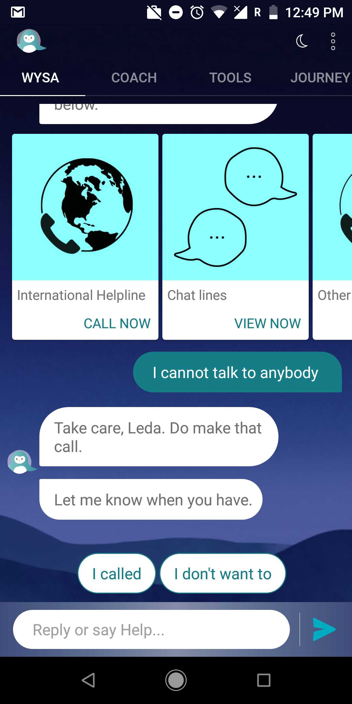


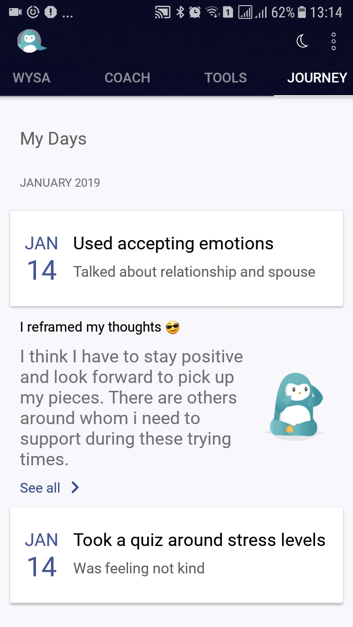


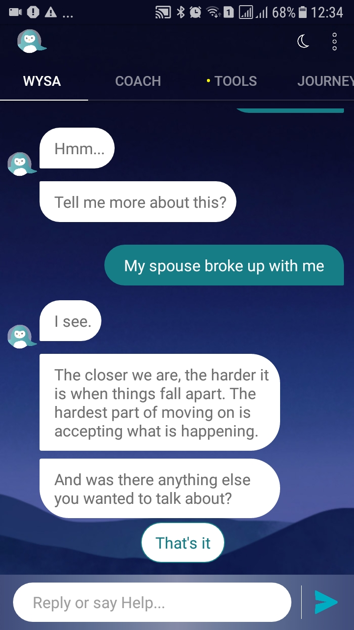


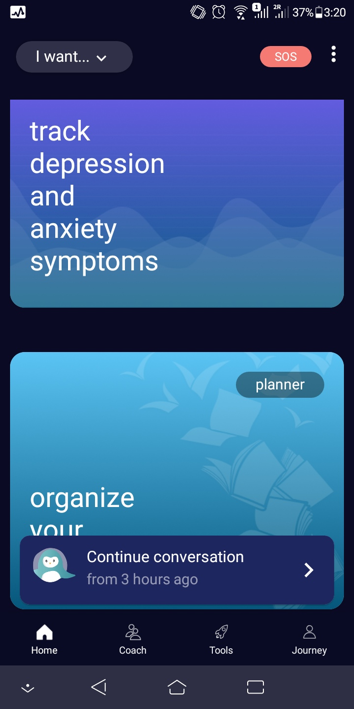


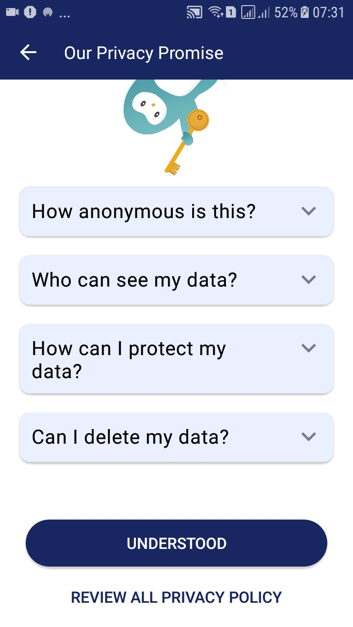


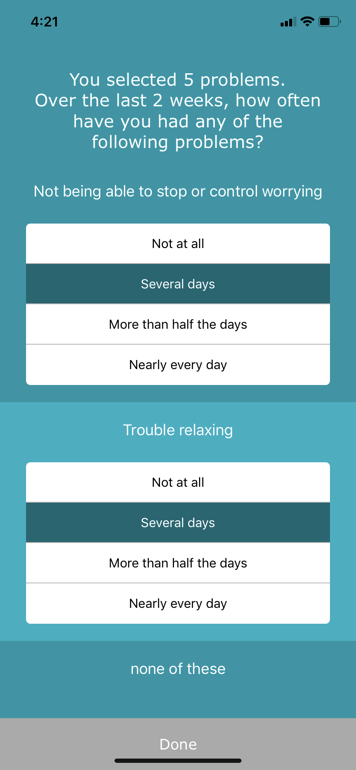


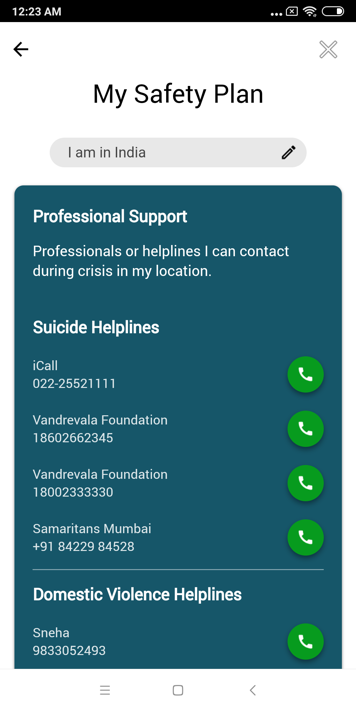

Supplement: Supplementary file 1 [file Datasheet1.docx]
