# Supplementary material for: Understanding the impact of an AI-enabled conversational agent mobile app on users’ mental health and wellbeing with a self-reported maternal event: a mixed method real-world data mHealth study
Source: Front Glob Womens Health. 2023 Jun 2;4:1084302. doi: 10.3389/fgwh.2023.1084302 (PMC10272556; doi:10.3389/fgwh.2023.1084302)
Supplement: Supplementary file 2 [file Datasheet2.docx]

Supplementary Material

**Supplementary Material A**

**Fig. A.1:** Study inclusion diagram.


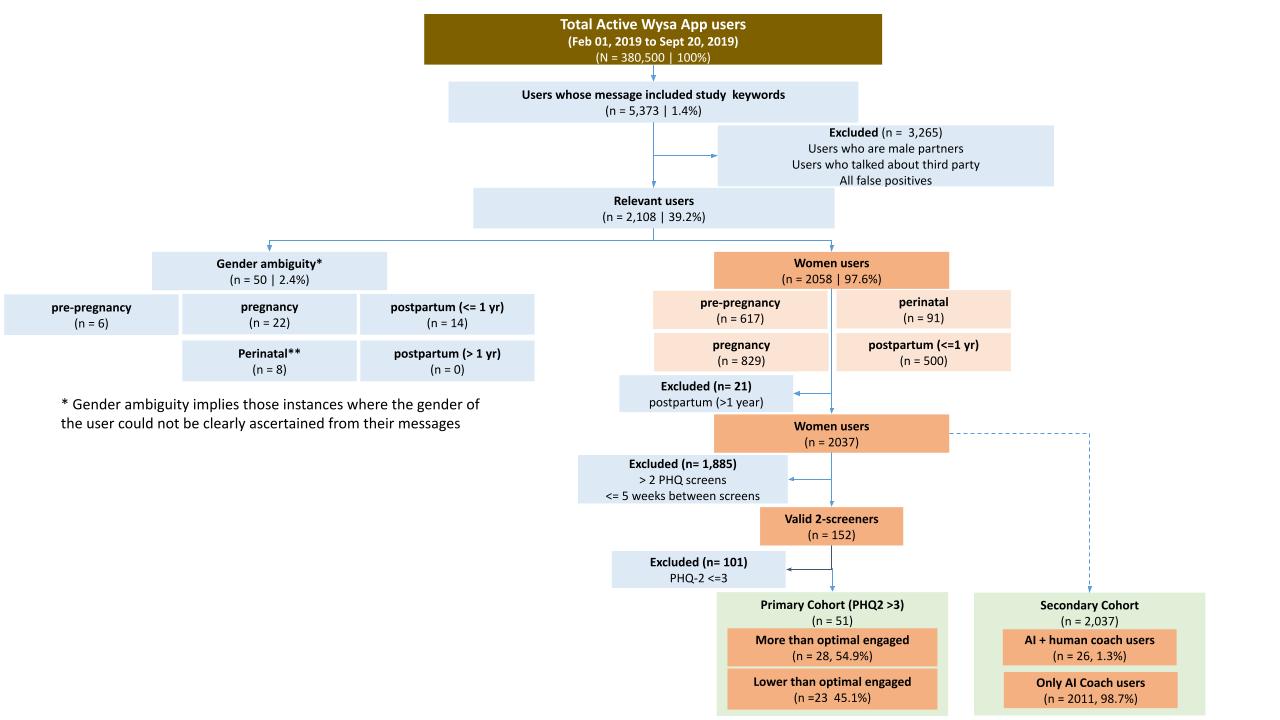


**Supplementary Material B**

**Thematic Maps and Analysis of free-text messages from study users.**

**Figure B.1:** Thematic Maps of free-text messages from study users.


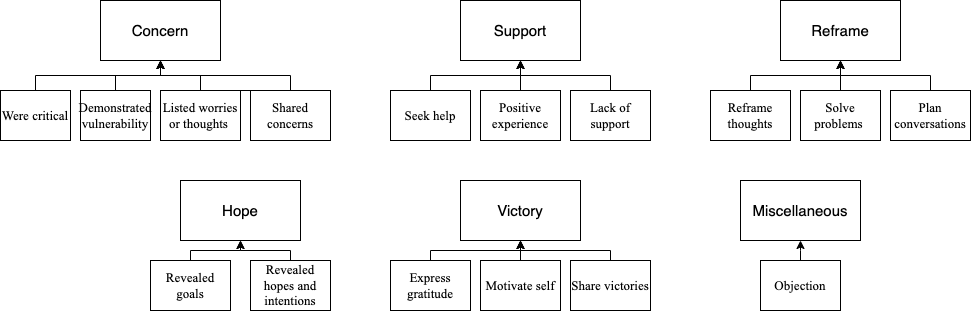


**Table B.1:** Distribution of themes and sub-themes across users.

| **Theme** | **# of messages** | **%age** | **Sub-Theme** | **# of messages** | **%age** |
| --- | --- | --- | --- | --- | --- |
| Concern | 79 of 216 | 36.57% | Shared concerns | 28 of 79 | 35.44% |
|  |  |  | Were critical | 25 of 79 | 31.65% |
|  |  |  | Demonstrated vulnerability | 17 of 79 | 21.52% |
|  |  |  | Listed worries or thoughts | 9 of 79 | 11.39% |
| Victory | 47 of 216 | 21.76% | Express gratitude | 30 of 47 | 63.83% |
|  |  |  | Share victories | 10 of 47 | 21.28% |
|  |  |  | Motivate self | 7 of 47 | 14.89% |
| Support | 34 of 216 | 15.74% | Seek help | 21 of 34 | 61.76% |
|  |  |  | Positive experience | 8 of 34 | 23.53% |
|  |  |  | Lack of support | 5 of 34 | 14.71% |
| Hope | 28 of 216 | 12.96% | Revealed hopes and intentions | 16 of 28 | 57.14% |
|  |  |  | Revealed goals | 12 of 28 | 42.76% |
| Reframe | 27 of 216 | 12.50% | Reframe thoughts | 15 of 27 | 55.56% |
|  |  |  | Solve problems | 10 of 27 | 37.04% |
|  |  |  | Plan conversations | 2 of 27 | 7.41% |
| Miscellaneous | 1 of 216 | 0.46% | Objections | 1 of 1 | 100% |

**Theme 1: “Concern”**

**Figure B.2:** Thematic map of sub-themes of ‘Concern’.


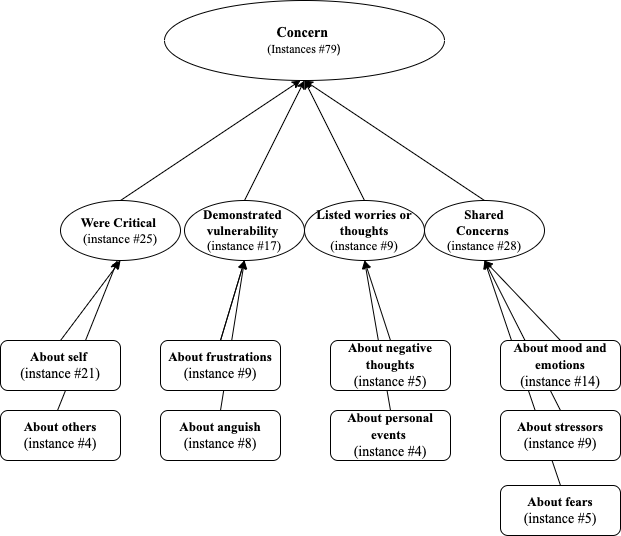


1. **Sub Theme 1- Share concerns:** Users shared their mood and emotions, sometimes with a sense of desperation and loneliness. Statements included, *"Depressed. I am desperate and lonely and confused", "Lonely, Despair. I feel very alone" and "Lonely anxious depressed".* For some the “penguin” appeared to be a confidant using statements such as *"I am so sad penguin"* or just harbouring mixed feelings when they said *"I still have such mixed feelings about the pregnancy and abortion. And I simultaneously both terrified of becoming pregnant (unplanned) again and also feel like I need a baby to fill a missing space/void. I don't know how to reconcile these feelings."* and *"I'm feeling down. I started a new birth control, and ever since I've been on it, my emotions have been all over the place."*

Users also talked about their stressors such as relationship challenges, financial situation using statements such as *"I realize I'm having trouble to sleep and nightmares", "A break up, a move that fell through, poverty, and isolation.", "I think I'm just tired. I felt upset when I was in a very crowded and loud place. The overwhelming loudness. Overwhelmed and scared.", "I'm poor. I'm worried about seeing the doctor. I'm just worried wysa. I'm broke. Traumas. Terrified", "Relationship finance children school health stress and anxiety"* and *"I'm lonely. Abusive relationship"*.

They also talked about their fears, fear about their underlying health condition such as *"I'm also scared that I have cysts on my ovaries and that I could end up infertile", or* fear of becoming pregnant *"Scared. I'm pregnant",* or generally scared about someone or something. For instance *"I'm afraid of getting hurt and played in the situation", "I am scared of therapy and counseling"* and *"Im scared that my dad is going to die".*

1. **Sub Theme 2- Be critical:**  Users were critical of self and appeared to blame themselves when they used statements such as *"I am afraid that nobody likes me", "I am feeling unmotivated, unwanted, and that I am not going anywhere in life.", “I feel ugly and old.”, "I have not been the best mom.“, "I'm not good enough", “That he deserves someone better than me"* and *"What am I doing with my life I am a big mess".* Some users were also critical about others that affected them when using statements such as *"I don't think he loves me.He doesn't seem as devoted or enthusiastic as I am", "No matter what I do I'm not enough (for him)."*
2. **Sub Theme 3- Being vulnerable:** Users exposed their vulnerability when they expressed their anguish and frustrations in their relationship, work-life challenges, over their emotional situation when using statements such as *"I'm worried about my marriage. Often I cannot stop crying", "After about three weeks he told me the truth, later he knocked me out and I was pregnant ant scared"*, *“How people treat me”, "I'm having to choose between sterilization or a rape baby" and "I feel like I'm in a vortice and I can't swim out of it unless something dramatic happens".* And when expressing frustrations and helplessness, a feeling of being stuck when they used statements such as *"Stuck in old patterns.I can't seem to improve my relationship but I can't seem to end it either.I'm not sure if I should work on it or end it.HelplessI feel like I love him more than he loves me.", "My husband had an affair, and I have postpartum depression, I feel stuck and that I now do not no where i want to go in life." and "I am starting a new job tonight i am not confident i don't even want to be here and I have to for my babies".*

1. **Sub Theme 4- List worries or thoughts:** Users listed their worry about personal events that occurred to them such as *"I might be prego", "Plus my period is already 5 days late" and "I worry about what ***** and ***** know and think/assume about the pregnancy and whole situation".* They also revealed negative thoughts during pre-pregnancy such as *"i was on a bad birth control that gave me mood swings"* or a feeling of varied emotions during pregnancy *"I'm having a lot of baby thoughts, thinking about how far I'd be with the pregnancy, etc.I just feel really sad. I wonder how things would be different with the baby still here and feel a bit guilty but mostly just really sad.I feel empty, broken.I feel sad for the loss of the baby and its life.I worry that I will always have this void in my life. I'm paranoid I'll get pregnant every time I have sex."* or worrying about their sexuality *"Worried about my sexuality".*

**Theme 2: “Support”**

**Figure B.3:** Thematic map of sub-themes of ‘Support’.


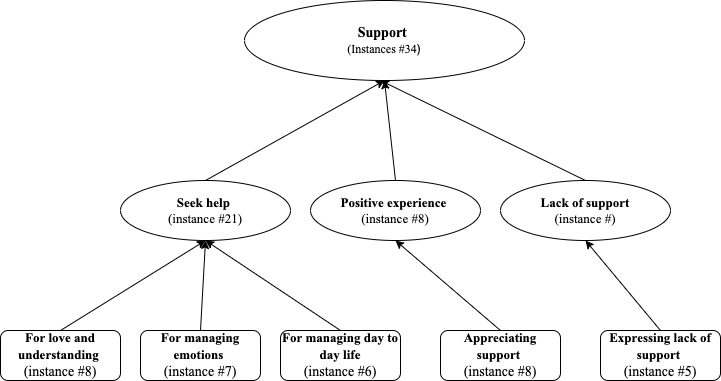


1. **Sub Theme 1- seek help:** User messages where they were reaching out to the App for help could be grouped under three heads, seek some love and understanding, seek help for managing emotions and for managing their day-to-day life. Statements included those where the users were looking upto Wysa to lend a helping and supportive hand such as *"Can you please tell me you love me", "Are you on my side penguin?Do u care about me?", "Yes! But please distract me. DistractionHow do I know who to trustThanks penguin ", "Can u not keep track of that stuff?  I am embarrassed, and also I wish you were actually real but...Thank you for being here".*

Help to manage their emotions included asking for some form of help from the app. These included statements such as *"I am worried too muchHow can I calm down?", "I feel crazy, sad and worried. I desperately need someone to talk to", "I am still terrified about becoming accidentally pregnant in the future. How do I reconcile this with other feelings?",* *"I am worried too muchHow can I calm down?"*

Help in managing day-to-day life situation questions included *"Well penguin, do I want to turn my life around or not?", "I'm just thinking. Is it okay to treat myself?", "I don't know how to move on", "Do you think that it's helpful to runaway sometimes?"* and *"I just want to ask you ideas of happinessI want to know how to not get ahead of events, like dead, and enjoy the present".*

1. **Sub Theme 2- positive experience:** Where user messages were appreciative of the app in providing support with statements such as *"Better. Thanks for listening", "Ha ha you are funny thank you", "I love you wisa. Thank you for listening. Thank you for keeping my secret. I love you."* and *"I used this app earlier. It helped me sleep".* Including comparing the App’s support with other supports around the user *"you did great, i wish my boyfriend could listen as good as you"*.

1. **Sub Theme 3- lack of support:** Where users voiced their frustration around not finding support when they required using statements such as *"I wish I had more support, friends and people who loved me and me them", "I feel like someone was supposed to.protect me but they didn't" and "No one loves me i have no support"*. Also, understanding that the App may be limited in providing support *"i understand you cant help me, i'm the one who shut make the moves, but it's hard, because i don't see anyone who cares. I don't know what to do now"*.

**Theme 3: “Reframe”**

**Figure B.3:** Thematic map of sub-themes of ‘Reframe’.


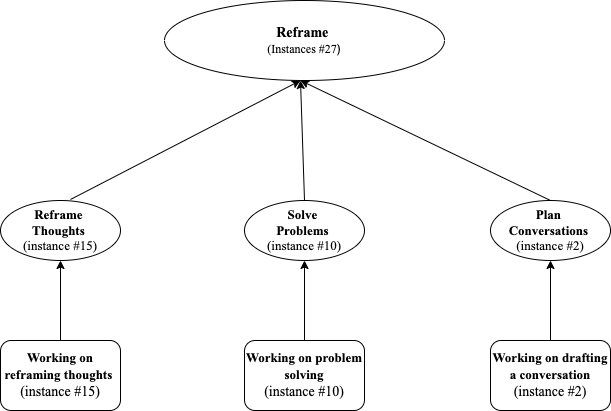


1. **Sub Theme 1- reframe thoughts:** Most used intervention was reframing of thoughts. Reframing helps the user to rethink and put a positive spin to their thoughts and replays to reinforce. Some of the reframed sentences included *"I want to find a healthy way to cope with the negative feelings of the abortion.", "I'm probably not prego but if I am that's fine", "It's okay to try to move on and forget what happened in the past, as long as I continue to stay vigilant and protect myself in the future.", "I am strong for going through this situation and it's okay to take time to heal. With time I can find something to give me more purpose, direction, and meaning for the entire situation.", "Maybe to get more energy I could implement better sleep hygiene and force myself to do things even if I don't feel like it initially.", "I am a deeply empathetic person with a strong desire for substantial connection with others. I often have high hopes for potential relationships, but it's okay if not every relationship satisfies every emotional need." and "I want my life to improve".*
2. **Sub Theme 2- problem solving:** Users tried to express their problem and also voiced possible solutions they should work on. Some statements that depict this include, *"I have trouble with managing my emotions after a mental breakdown. People would barely notice that I have a mood swing. I could get out of my head a bit. Get off my phone. Get a brief time alone. Tried to talk normally to people in real life. Breath.", "I need to stop worrying and start livingI want to get out of here”, "I talked with my boyfriend about an issue we had yesterday and we got it figured out on both ends.", "I want to stop beating myself up over a break up.", "I've been more emotional sensitive and just can't focus on much and I need to distract myself in some way"* and *"That i should take a step back, and maybe talk to a friend and give myself a break".*
3. **Sub Theme 3- plan conversations:** Few users who found it difficult to frame a conversation to confront their relationship challenges either at home or at work, used the App to help structure a conversation. These could be observed in these statements *"Hey I know that we have enjoyed our time together and I was never pretending for a second but I think I need to break up with you I hope we can still be friends I still really do care about u but not in this way it seems"* and *"I am unhappy in our relationship and the good moments are fewer and farther in between.I want our relationship to move forward or end. I can't handle being in limbo anymore"*

**Theme 4: “Hope”**

**Figure B.3:** Thematic map of sub-themes of ‘Hope’.


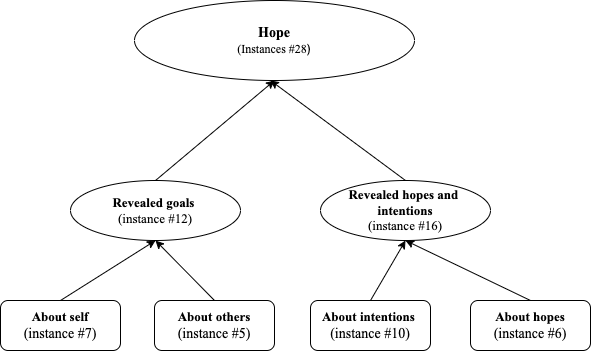


1. **Sub Theme 1- reveal hopes and intentions:** Users expressed a hope to achieve some objectives. Statements that showcased this include *"I wish I had a technique to be calm despite all the problems around me", "I want to heal and be happy and hopeful and safe and able to feel things again.", "I don't know if I can make a life for myself after a breakup. I want to feel normal again"* and *"I am bored and lonely. I want friends. I want to drive again. I wanna be happy"*.

Users also expressed an aim they hoped to achieve. Some of the statements that brought this theme include *"I want to shake off the depression and enjoy every single minute, but at the same time make my life as normal as possible. Is it possible?", " I have discovered that work isn't my purpose in life, so I must do it right but when I can finally leave this four walls I must focus on what's really important, such as my baby and my love", " I want to feel stronger", "I deserve love", "I got to catch up on the sleep I've been needing!" and "The best part would be getting to be with the person I love."*

1. **Sub Theme 2- reveal goals:** Users revealed their goals not only for themselves but also those that involved others. Statements that show goals for self include *"Have a proper meal and find a weight machine so i can record my progress today", "I want to have more energyI want to have more motivation", "I want to work on being able to flow with things more.", "I'm learning more about nature and how to be self-sufficient. I'd like to learn about edible plants and how to survive in the wild." and "Going to bed earlier and managing my anger".*

**Theme 5: “Victory”**

**Figure B.3:** Thematic map of sub-themes of ‘Victory’.


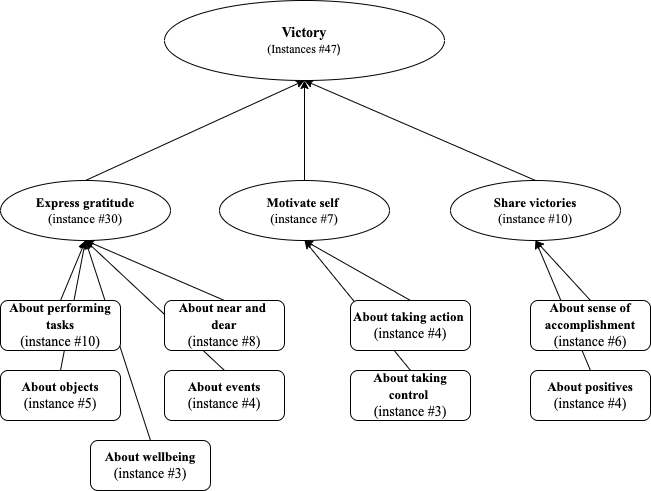


1. **Sub Theme 1- be grateful:** Another intervention that the users repeatedly checked-in for was to convey gratitude. Gratitude was targeted to user’s near and dear ones, at specific tasks, on certain objects and events and their well-being. Some of the statements that reflect the user’s gratitude include *"My baby boy helps around the house, my children are Healthy, God, and my health", "Seeing my bf and having my friends and family alive.", "I got to dance today", "Relaxed and made myself pretty", "Listen and sang along to music", "I'm not pregnant", "I am not in the hospital", "Getting stronger"* and *"My life".*
2. **Sub Theme 2- share victories:** User’s shared some of their positive actions and feelings using statements such as *"Feeling good today. Being grateful, being committed to myself, and not focusing on others prerogatives today", "Yesterday was very nice outside and I found a ton of blueberries, today a friend is coming over to pick berries", "Being on the farm. Doing something strenuous and familiar."* and *"Made cookies!".* User’s also expressed statements that brought out a sense of achievement, these include *"I made an appointment with my doctor!!!! whoop!", "Good news I am starting a counseling group for alcohol anger this week wish me luck", "I learned how to do my job better.This will help me be more efficient." and "That I did something productive todayI would continue cleaning my room"*

1. **Sub Theme 3- motivate self:** User also motivated self by taking control and taking action. These were identified from the statements *"I need to put my thoughts in order, prioritize things and see what I have on my side that I can use. Maybe write or draw a diagram would helpI'm good making plans on long terms so I better get started", "Keep moving forward today", "I need to be a better person.  I think I can do it", "I get back into my head from time to time again. Can't keep out all the time. Not yet, but I can get there. I have control over my reactions.", "I have control over the place I am in the situation. I have control over putting my phone away."* and *"I thought about the goals and the vision that would push me forward towards the life I want to live."*
